# Hyperglycemia Changes Expression of Key Adipogenesis Markers *(C/EBPα and PPARᵞ)*and Morphology of Differentiating Human Visceral Adipocytes

**DOI:** 10.3390/nu11081835

**Published:** 2019-08-08

**Authors:** Ewa Świderska, Marta Podolska, Justyna Strycharz, Marzena Szwed, Halina Abramczyk, Beata Brożek-Płuska, Adam Wróblewski, Janusz Szemraj, Ireneusz Majsterek, Józef Drzewoski, Agnieszka Śliwińska

**Affiliations:** 1 Department of Medical Biochemistry, Medical University of Lodz, 92-215 Lodz, Poland; 2 Department of Internal Medicine, Diabetology and Clinical Pharmacology, Medical University of Lodz, 92-213 Lodz, Poland; 3 Department of Thermobiology, Faculty of Biology and Environmental Protection, University of Lodz, 90-236 Lodz, Poland; 4 Laboratory of Laser Molecular Spectroscopy, Lodz University of Technology, 93-590 Lodz, Poland; 5 Department of Clinical Chemistry and Biochemistry, Medical University of Lodz, 90-647 Lodz, Poland; 6 Central Teaching Hospital of the Medical University of Lodz, 92-213 Lodz, Poland; 7 Department of Nucleic Acids Biochemistry, Medical University of Lodz, 92-213 Lodz, Poland

**Keywords:** visceral preadipocytes, adipocytes, adipogenesis, PPARγ, C/EBPα, miR-34a-5p, miR-137-3p, hyperglycemia, diabetes

## Abstract

Disturbances in adipose tissue significantly contribute to the development of metabolic disorders, which are connected with hyperglycemia (HG) and underlain by epigenetics-based mechanisms. Therefore, we aimed to evaluate the effect of hyperglycemia on proliferating, differentiating and maturating human visceral pre/adipocytes (HPA-v). Three stages of cell culture were conducted under constant or variable glycemic conditions. Adipogenesis progress was assessed using BODIPY 505/515 staining. Lipid content typical for normal and hyperglycemic conditions of adipocytes was analyzed using Raman spectroscopy and imaging. Expression of adipogenic markers, PPARγ and C/EBPα, was determined at the mRNA and protein levels. We also examined expression of miRNAs proven to target PPARγ (miR-34a-5p) and C/EBPα (miR-137-3p), employing TaqMan Low-Density Arrays (TLDA) cards. Hyperglycemia altered morphology of differentiating HPA-v in relation to normoglycemia by accelerating the formation of lipid droplets and making their numbers and volume increase. Raman results confirmed that the qualitative and quantitative lipid composition under normal and hyperglycemic conditions were different, and that the number of lipid droplets increased in (HG)-treated cells. Expression profiles of both examined genes markedly changed either during adipogenesis under physiological and hyperglycemic conditions, orat particular stages of adipogenesis upon chronic and/or variable glycemia. Expression levels of PPARγ seemed to correspond to some expression changes of miR-34a-5p. miR-137-3p, whose expression was rather stable throughout the culture, did not seem to affect C/EBPα. Our observations revealed that chronic and intermittent hyperglycemia change the morphology of visceral pre/adipocytes during adipogenesis. Moreover, hyperglycemia may utilize miR-34a-5p to induce some expression changes in PPARγ.

## 1. Introduction

Adipose tissue (AT) surrounding internal organs, termed “visceral fat”, has been recently suggested as a potential site of initiation formetabolic disorders [1,2,3]. Overgrowth of visceral fat depots is correlated with increased secretion of pro-inflammatory cytokines, whichfurther raise the risk for developing type 2 diabetes mellitus (T2DM) [4,5]. AT growth is mostly connected with the promotion of adipogenesis (ADG), which involves the formation and expansion of AT with preadipocytes (pAds) that start to proliferate and differentiate into mature adipocytes (Ads), thereby storing the fat [6]. The process consists of two stages: at first, pluripotent mesenchymal stem cells (MSCs) differentiate into pAds under the influence of external signals (the commitment phase);then, pAds exposed to hormones and growth factors may differentiate into mature Ads, which are associated with the induction of the MAPK pathway and, consequently, the expression of several ADG-related transcription factors [7,8]. These include the CCAAT/enhancer binding protein (C/EBP) gene family, peroxisome proliferator activated receptor-γ (PPARγ), sterol regulatory element binding protein 1 (SREBP1), DNA-damage-inducible transcript 3 (DDIT3) and the Kruppel-like factor (KLF) gene family.

It was found that ADG occurs mainly via the action of PPARγ and C/EBPα [9,10]. PPARγ is a member of the nuclear receptor family of PPARs. Two isoforms of PPARγ have been found—PPARγ1 and PPARγ2—which generally do not differ functionally. PPAR-γ is regarded as a mostly AT-specific gene with a relatively low level of expression in other tissues [11,12,13]. The research revealed a substantial contribution of PPARγ in obesity, diabetes, atherosclerosis and neoplasms. Another studied transcription factor, C/EBPα, was found to inhibit cell growth via inactivation of cyclin-dependent kinases CDK2 and CDK4, induce adipogenesis in mice and serve as an essential factor for 3T3-L1 pAds differentiation [14]. Furthermore, it was reported that ahigh level of the C/EBPα gene maintains Ads in their fully differentiated state [2,14,15]. It is known that PPAR-γ and C/EBPα may induce the expression of several genes associated with insulin sensitivity (glucose transporter type 4(GLUT4)), lipogenesis (fatty-acid binding protein (FABP)), lipolysis (lipoprotein lipase (LPL)) as well as some adipocytokines (adiponectin and leptin) [7].

Recently, it has been suggested that alterations in gene expression during ADG could be triggered by epigenetic regulation [16,17,18,19]. Epigenetic modifications involve several alterations during transcriptional and translational processes and include mainly the reversible DNA methylation and histones methylation and acetylation, along with microRNA (miRNA) interference. miRNAs complementary to specific mRNA sites may inhibit and/or degrade targeted genes, therefore impacting the level of specific proteins [20,21]. Deregulation of miRNAs is suggested to be an important element of the pathophysiology of various diseases, including metabolic disorders.

Although ADG has been extensively studied in recent years, a question about the precise mechanisms governing this process still remains unanswered, especially in the context of T2DM. Hyperglycemia (HG), as an integral element of the T2DM pathogenesis and progress, was found to induce adipogenic processes in adipose- and muscle-derived stem cells (ADSCs, MDSCs) [22], osteosarcoma cells [23] and primary rat osteoblasts [24]. However, HG was not sufficient to potently accelerate ADG and increase TG content inhuman subcutaneous tissue-derived pAds in comparison with normoglycemia (NG) [25]. The impact of HG on the rate of proliferation or senescence of subcutaneous and visceral pAds isolated from diabetic patients was evaluated as inconsistent in several studies [26].

Taking these studies into account, we asked whether: (i) HG changes the morphology and composition of lipid droplets in visceral p/Ads not only during the mere process of differentiation, but also their proliferation and maturation; (ii) potentially introduced changes are reversible upon variable glycemic conditions; and (iii) all these processes are accompanied by expression changes of ADG-related molecules. Therefore, the aim of the present study was to explore the impact of chronic or intermittent HG on visceral ADG, and to elucidate the influence of glycemia on the gene expression and protein level of two crucial markers of this process, PPARγ and C/EBPα. Furthermore, in order to establish whether HG may affect the expression of these adipogenic markers via utilizing epigenetic mechanisms, we evaluated the expression levels of miR-34a-5p and miR-137-3p, which have been proven to be negative regulators of PPARγ and C/EBPα, respectively [27,28].

## 2. Materials and Methods

### 2.1. Cell Culture

Human visceral pAds (HPA-v) were cultured as described in [29]. In general, cell culture was divided into three stages: proliferation (5 days), differentiation (12 days) and maturation (6 days). During the first stage of culture, pAds were maintained in preadipocyte medium (PAM). After reaching full confluence, pAds were cultured in preadipocyte differentiation medium (PADM). Mature Ads were maintained in adipocyte medium (AdM). Then, after completion of each stage, cells were harvested and the cell pellet was processed specifically for further experimental aims. Cells and reagents for cell cultures were obtained from ScienCell Research Laboratories (Carlsbad, CA, USA). Three independent experiments of cell culture were conducted.

### 2.2. Glycemic Conditions

In order to obtain hyperglycemic conditions, the cells were grown in full medium supplemented with glucose (d-(+)-Glucose, Sigma-Aldrich) to obtain a final concentration of 30mM. As a result, we designed 14cell culture variants (Table 1).Variants cultured in chronic NG/HG were cells maintained in medium with a 5.5 mM/30 mM concentration of glucose during all stages of adipogenesis (N, NN, NNN/H, HH, HHH). Variants cultured in intermittent glycemia were maintained in both glucose concentrations (e.g., the NHH variant reflected cells cultured in 5.5 mM of glucose during one stage of culture and 30 mM in the next two stages). While interpreting the results, all variants treated chronically with HG (H, HH, HHH) were designed to reflect a diabetic patient, while all three variants maintained in NG (N, NN, NNN) were to mimic a healthy and normoglycemic subject. Other variants were designed in order to: (i) examine the relevance of exposure to HG at each stage of ADG, (ii) select the ADG stage critical for introducing changes typical of diabetic phenotypes, and (iii) observe the effect of glycemianormalization. Therefore, intermittent HG variants were used to mimic patients with glycemia fluctuations.‘N’ and ‘H’ were introduced to mark normoglycemic and hyperglycemic culture conditions, respectively.

### 2.3. Lipid Droplets Staining by BODIPY 505/515.

pAds and Ads cultured under different glycemic conditions were stained with BODIPY 505/515 fluorescence probe at specific time points (during proliferation, differentiation and maturation), as well as after achievement of the three culture stages. Briefly, cells were incubated for 30 min at 37°C with 20 µM of BODIPY 505/515 (Life Technologies, Eugene, OR, USA) diluted in PBS. Fluorescence emitted by stained cells was then observed in an inverted fluorescence microscope (Olympus IX70, Tokyo, Japan, magnification ×400).

### 2.4. Raman Spectroscopy and Imaging

To explore the morphological and biochemical compositions of p/Ads using Raman spectroscopy and imaging, Raman images and spectra were recorded using a confocal Raman microscope, the WITec alpha300 RSA (Germany). The Alpha300 RSA consists of an Olympus microscope coupled via the fiber (50 μm core diameter) with a 300-mm monochromator (Princeton Instruments Acton SP23000, 300-mm triple grating Monochromator/Spectrograph) and a thermoelectrically cooled Charge-Coupled Device CCD camera, the ANDOR Newton DU970N-UVB-353 (EMCCD chip with 1600 × 200 pixels) operating in the standard mode at −60 °C (option: full vertical binning). To obtain high spatially resolved Raman spectra, the 40× objective (NICON CFI Plan Fluor C ELWD 40×:N.A. 0.60, W.D. 3.6–2.8 mm; DIC-M, C.C.0-2) was used in combination with a piezo stage. All spectra and imagings were recorded using Second Harmonic Generation (SHG) of the Nd:YAG laser (532 nm). The analysis of Raman spectra and imagings was performed using WITec Project 4 Plus.

### 2.5. RNA Isolation and mRNA Expression Profiling

Each culture variant comprising approximately 6 × 10^6^ cells was harvested, lysed and homogenized using an AllPrep DNA/RNA/Protein Mini kit (QIAGEN, Hilden, Germany). Briefly, the cell pellet was disrupted by the addition of RLT buffer with B-mercaptoethanol. Lysed cells were then passed several times through a 20-gauge needle (0.9 mm diameter) fitted to an RNase-free syringe, followed by RNA isolation according to the manufacturer’s instructions. Concentration and purity of RNA was measured with a Synergy HT microplate reader (Biotek). Isolated RNA was then converted into cDNA with a High Capacity cDNA Reverse Transcription Kit (Applied Biosystems, Foster City, CA, USA). Quantitative Real-time PCR was performed using TaqMan Gene Expression Master Mix and TaqMan Gene Expression Assays: s00234592_m1 for PPARγ and Hs00269972_s1 for C/EBPα (Applied Biosystems, Foster City, CA, USA). The real-time PCR program was as follows: hold (50°C, 2 min), hold (95°C, 10 min), 40 cycles (95°C, 15 s), hold (60°C, 1 min). For real-time PCR data normalization, we used the 2^−ΔCt^ method and the arithmetic average of Ct values obtained for RLPLO (ribosomal protein lateral stalk subunit P0) and UBC (ubiquitin C). Selection of the most relatively stable genes involved expression-profiling of 16 reference genes for each cell culture variant with the equalized RNA amount for reverse transcription using the above mentioned reagents and TaqMan Array Human Endogenous Control Plates (Applied Biosystems, Foster City, CA, USA) in accordance with the protocol. In RefFinder [30], we performed several analyses involving groups of variants while taking into account: (i) ADG in constant glycemia, (ii) three stages of differentiation and (iii) planned sets of culture variants to be compared. This was followed by the integration of all obtained results.

### 2.6. Protein Isolation and Enzyme-Linked Immunosorbent Assay (ELISA)

Total protein was isolated from the cell culture using RIPA buffer with Pierce mini protease and phosphatase inhibitors tablets (Thermo Scientific, Rockford, IL, USA). Briefly, cells were trypsinized and washed three times with DPBS, followed by incubation on ice for 15 min and centrifugation (15,000 rpm, 4°C, 15 min). Supernatant was subjected to measurement of protein concentration with a Protein Determination Kit (Caymann, Ann Arbor, MI, USA) and frozen in −80°C for further analyses. ELISA assays were performed for PPARᵞ and C/EBPα according to the standard attached instruction (SEA886Hu-PPARᵞ, SEA644Hu-C/EBPα, Cloud-Clone Corp, Katy, Texas, USA ).

### 2.7. miRNA Isolation and Expression Profiling

Detailed information regarding miRNA isolation and expression profiling has been described previously [29]. Briefly, miRNA was isolated using an miRVANA Isolation Kit (Applied Biosystems, Vilnius, Lithuania) then reverse transcribed with reagents from Applied Biosystems (Foster City, CA, USA). Expression profiling was preceded by screening analysis and performed with TaqMan Low-Density Arrays (TLDA) cards (Applied Biosystems, Foster City, CA, USA) in a 7900HT Fast Real-Time PCR System (Applied Biosystems, Foster City, CA, USA). Data normalization was performed using the 2^−∆Ct^ method with the arithmetic average of Ct values for U6 and let-7b-5p, as supported by RefFinder [30]. Assay IDs (Applied Biosystems) used in this experiment were as follows: hsa-miR-34a-5p (000426), hsa-miR-137-3p (001129), hsa-let-7b-5p (002619) and U6 (001973). Selection of miRNAs, which are presented herein, was based not only on screening analysis but also on bioinformatics analysis and literature search.

### 2.8. Groups Comparisons and Statistical Analysis

Analysis of expression changes along with statistics (GraphPad Prism 6.0) were performed in a way allowing for the examination of the impact of one variable only—either ADG or HG (single, double and chronic exposure to HG vs NG, as well as stage-specific changes such as the comparison of an NHH variant to NHN and NNH, or HH vs NH, etc.). A two-tailed t-test was exploited to test the statistical significance between every two mean values. To evaluate statistical significance in multiple pair-wise comparisons (ADG in NG or HG) we used one-way ANOVA with a post-hoc Tukey test. For all of the statistics, *p* ≤ 0.05 was regarded as significant.

## 3. Results

### 3.1. Morphological Changes of p/Ads Cultured in Normoglycemic and Constant/Intermittent Hyperglycemic Conditions

**Figure 1:**
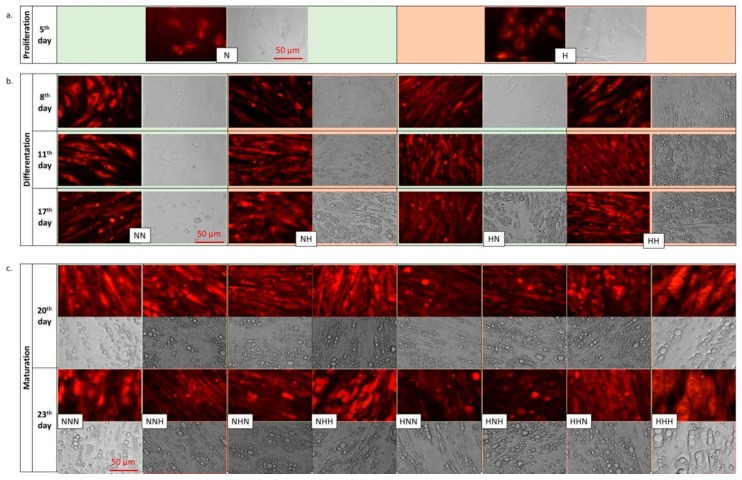
Morphological changes of human visceral p/Ads during ADG undernormoglycemic and chronic/intermittent hyperglycemic conditions, applied throughout the entire period of culture. Cells stained (**a**) at the termination of the proliferation stage, (**b**) during and at the termination of the differentiation stage and (**c**) during and at the termination of the maturation stage. Cells were stained with BODIPY 505/515 and analyzed under an inverted fluorescence microscope (Olympus IX70), with a magnification ×400 underthe conditions of fluorescence (upper panel of NG or HG, respectively) or phase contrast (lower panel of shown photos).

pAds cultured in both NG and HG resembled fibroblasts at the proliferation stage(Figure 1a). However, several days after starting, differentiation cells exhibited altered morphology and accumulated lipid droplets (Figure 1b). Observation of morphological changes during the whole cell culture suggested that HG significantly accelerates differentiation and maturation of human visceral pAds in comparison with pAds differentiating in medium with standard glucose content (NG) (Figure 1b). This was evidenced by an increased number of lipid droplets in cells cultured in HG that had already appeared by the eighth day of culture (third day of differentiation). Moreover, lipid droplets were bigger in size. The most relevant changes were observed for the cells cultured in chronic HG during the whole process of ADG (Figure 1c). The content, as well as the size of lipid droplets in this variant (HHH), were the highest among all of the variants cultured.

During the differentiation stage, cells treated with single or double hyperglycemic hits(NH, HN, HH) were markedly different from cells cultured in chronic NG. Whereas first lipid droplets in the NN variant appeared on the 17th day of culture, in other variants they were already visible by the eighth (HH) or 11th day (HN, NH) of culture (Figure 1b). Considering the mature Ads, it seemed that all double HG-treated variants showed alarger size and increased number of lipids than the variants with single HG hits(Figure 1c); however, this requires further testing for quantitative confirmation. Altogether, this data may imply that the morphological changes of human visceral Ads during ADG were strongly dependent on the length of the period of HG exposure rather than an association with stage-specific events.

### 3.2. Raman Spectroscopy and Imaging

**Figure 2:**
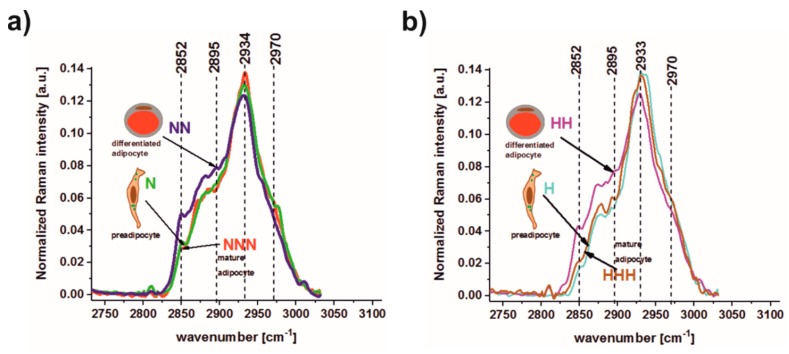
The average Raman spectra typical for the high-frequency region recorded for pAds, differentiated Ads and mature Ads under (**a**) normoglycemic (N, green line; NN, blue line; NNN, red line) and (**b**) hyperglycemic conditions (H, turquoise line; HH, purple line; HHH, brown line). Arrows are used to indicate which spectrum corresponds to a given type of adipocyte.

To monitor the chemical compositions of pAds (N, H), differentiated Ads (NN, HH) and mature Ads (NNN, HHH) we also applied Raman spectroscopy and imaging. Raman spectroscopy analyzes vibrations of molecules based on inelastic light scattering. The difference in energy of an incident and scattered photons delivers the information about molecule vibrations and the structure of chemical constituents. Each molecule (lipid, protein, carbohydrate, etc.) has itsown set of vibrational peaks, which can be monitored by Raman spectroscopy and imaging. Figure 2 shows the average Raman spectra typical for p/Ads in normoglycemic (a) and hyperglycemic (b) conditions.

**Figure 3:**
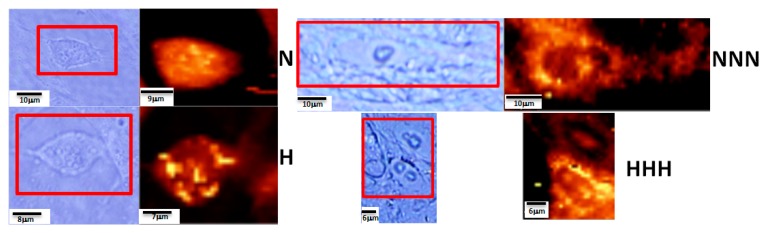
Microscopy images and the respective Raman images of pAds (N, H) and mature Ads (NNN, HHH) for intracellular structures: lipid structures (triacylglycerides (TAGs)), fatty acids (FAs) and cholesterol esters (CEs), (2840–2900 cm^−1^).

As shown in Figure 2, the Raman spectra in the high frequency region are sensitive to the degree of cell maturity. The main differences for N, NN, NNN, H, HH, and HHH can be seen inthe Raman peaks at 2852, 2895, 2934, 2970 cm^−1^. As we have mentioned above, each peak observed in Raman spectra can be associated with the functional groups typical of a sample’s different chemical constituents. It has been proven that the peak at 2852 cm^−1^ is typical oflong hydrocarbon chains, particularly in lipids, and corresponds to the C–H symmetric stretching vibrations of the methylene CH2 groups; the peak at 2895 cm^−1^ is typical for R3C–H stretching bands in proteins [31,32,33]; the peak at 2934 cm^−1^ corresponds to symmetric stretching C–H vibrations of the O=C–CH3 acetyl groups; while the peak at 2970 cm^−1^ corresponds to the antisymmetric stretching vibrational mode of the CH3 methyl group, which is contained in proteins, lipids and methylated DNA [32]. As shown in Figure 2a, the intensity of the peak at 2852 cm^−1^ increased for differentiated Ads (NN) compared to pAds (N), but returned to the original level for mature Ads (NNN). This observation confirms that the internal composition of a lipid fraction depends on Ads’ maturity. This was also indicated by the intensity of the peak 2895 cm^−1^, for which the highest result was observed for differentiated Ads (NN), and comparable intensities were noticed for pAds (N) and mature Ads (NNN). For the peak at 2970 cm^−1^, the intensity at first decreased from N to NN Ads, then again increased from NN to NNN cells. Such a relationship shows that methylation of lipids and proteins depends on Ads’ maturity levels, and that Raman spectroscopy can be used to monitor methylation status. The same Raman measurements were performed for Ads under hyperglycemic conditions. Peaks at 2852, 2895 and 2970 cm^−1^ for p/Ads cultured in HG were also related to cellular maturity level (Figure 2b), showing similar tendencies for p/Ads cultured in NG.

p/Ads can be monitored also by Raman imaging. Raman maps created in imaging mode allowed us to visualize the lipid structures including lipid droplets. Figure 3 presents the Raman imaging for N, H, NNN and HHH Ads. Raman imaging results presented in Figure 3 confirm that the number of lipid droplets was higher for Ads cultured under chronic HG.

### 3.3. Evaluation of Expression Profile of C/EBPα and Mir-137-3p in p/Ads Cultured in Normoglycemic and Constant/Intermittent Hyperglycemic Conditions

#### 3.3.1. Expression Changes Evoked during ADG in NG and HG

**Figure 4:**
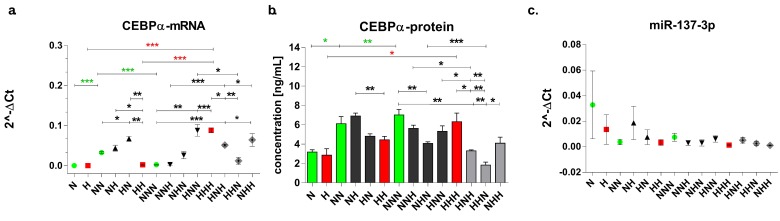
C/EBPα (mRNA and protein) and mir-137-3p expression profile during adipogenesis in chronic/intermittent normoglycemic (N) and hyperglycemic (H) conditions. (**a**) The relative levels of mRNA expression and (**b**) protein concentration of C/EBPα and (**c**) relative levels of miR-137-3p expression evaluated after completion of proliferation, differentiation and maturation of visceral cells. Data are expressed as mean ± SEM. Differences in expression levels between two particular culture variants were evaluated using a two-tailed t-test. One-way ANOVA with a post hoc Tukey test was used for calculation of statistical significance of changes observed during ADG in NG (NN vs N, NNN vs N, NNN vs NN) and HG (HH vs H, HHH vs H, HHH vs HH). *** *p* ≤ 0.001; ** *p* ≤ 0.01; * *p* ≤ 0.05.

The levels of mRNA and protein expression of C/EBPα and mir-137-3p during the process of ADG in chronic NG and HG are presented in Figure 4. We found that mRNA expression of C/EBPα during ADG in NG increased in differentiated Ads (NN vs N), and then decreased in mature Ads (NNN vs NN). The protein level of C/EBPα increased gradually during ADG (NN vs N; NNN vs N/NN). Regarding ADG in hyperglycemic conditions, we observed that mRNA expression of C/EBPα did not change in differentiated Ads (HH vs H), but significantly increased in mature Ads (HHH vs H/HH). The protein level increased with the progress of ADG, reaching the highest level in mature Ads. The expression of miR-137-3p during both normo-and hyperglycemic ADG did not show significant differences between particular stages. In summary, mRNA level reached a peak after the maturation stage, which was consistent with a gradual increase of C/EBPα protein concentration during ADG in HG. Cells differentiated in NG also exhibited a gradual protein concentration rise during culture, yet this was not reflected in mRNA expression, which declined after the maturation of the cells.

#### 3.3.2. Chronic and Intermittent HG-Triggered Expression Changes

Considering stage-specific changes, we observed that at the proliferation stage there were no differences between H and N variants both at mRNA and protein levels (Figure 4). At the differentiation stage, the HH variant showed a much lower expression of mRNA and protein than the NN one. Mature Ads cultured in chronic HG almost equalized the level of protein to cells cultured in NG (HHH vs NNN), while mRNA data indicated an upregulation of C/EBPα. To summarize, although the pattern of C/EBPα protein expression for NG- and HG-cultured p/Ads was similar, some differences were found at the mRNA level. We detected no changes of miR-137-3p in HG-versusNG-cultured cells.

As shown in Figure 4, in differentiated cells we observed significant upregulation of mRNA levels of C/EBPα in response to single HG stimulation (NH, HN) in comparison with the HH variant (Figure 4a). On the other hand, the NH variant showed an expression level similar to those found in differentiated Ads in the chronic NG (NN), suggesting that at this stage the introduction of HG did not affect the expression of C/EBPα. C/EBPα protein in the NH variant exhibited comparable protein expression to the NN cells, which was similar to the results obtained at the mRNA level. In contrast, protein expression detected in the HN cells was more similar to one observed in the HH cells. Expression of mir-137-3p at this stage remained stable, and did not seem to affect mRNA.

Considering single HG hits versus NG (NNN) in mature Ads, we did not observe any C/EBPα mRNA expression change when HG was introduced only at the maturation stage (NNH), or only at the differentiation stage (NHN). Moreover, among the double HG-hit variants, no difference between the HHN versus the NNN variant was observed. These four variants (NNN, NNH, NHN, HHN) formed a shared expression cluster. Interestingly, we noticed that another expression cluster was formed from HNN, NHH and HHH variants. Taking this data into account, these results may imply that the mRNA expression level of C/EBPα is more dependent on stage-specific events than the length of the period of hyperglycemic exposure. At the protein level, all variants cultured in intermittent HG showed a decrease in expression in relation to chronic NG and HG. Among single HG-hit variants, only comparison of the NHN to NNN showed a significant reduction in C/EBPα protein concentration. In case of double HG-hit variants, reduction was more intense and all variants were significantly downregulated in comparison to the NNN variant. What is more, both HNH and HHN variants were significantly downregulated when compared to the HHH variant. In mature Ads, mir-137-3p expression did not differ between cells treated with single or double hyperglycemic stimulation compared to both NNN and HHH.

To conclude, the data thus far suggests that changes induced by HG are more dependent on which stage the HG was introduced at than the length of the period of HG exposure. Moreover, proliferation (for mRNA) and differentiation (for protein) were stages at which HG had the most pronounced impact on C/EBPα expression. We found similarities in patterns between the expression of C/EBPα mRNA and protein, especially in variants treated with double HG hits. However, there were some differences between mRNA and protein, mostly seen in mature Ads treated with single HG hits. Thus, we hypothesize that there must be some regulatory mechanism other than mir-137-3p, as this molecule remained stable in all variants with intermittent HG.

### 3.4. Evaluation of Expression Profile of PPARγ and Mir-34a-5p in p/Ads Cultured in Normoglycemic and Constant/Intermittent Hyperglycemic Conditions

#### 3.4.1. Expression Changes Evoked during ADG in NG and HG

**Figure 5:**
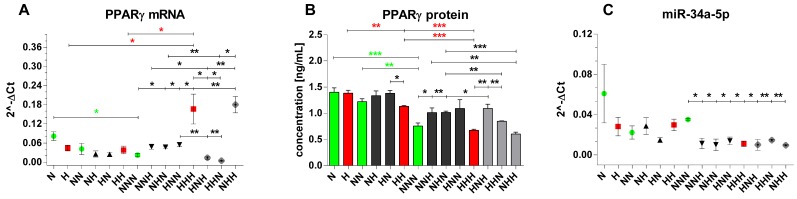
PPARγ (mRNA and protein) and mir-34a-5p expression profiles during adipogenesis in chronic/intermittent normoglycemic (N) and hyperglycemic (H) conditions. (**a)** The relative levels of mRNA expression and (**b**) protein concentration of PPARγ, as well as (**c**) the relative levels of miR-34a-5p expression evaluated after completion of proliferation, differentiation and maturation of visceral cells. Data are expressed as mean ± SEM. Differences in expression levels between two particular culture variants were evaluated using a two-tailed t-test. One-way ANOVA with a post hoc Tukey test was used for calculation of statistical significance of changes observed during ADG in NG (NN vs N, NNN vs N, NNN vs NN) and HG (HH vs H, HHH vs H, HHH vs HH). *** *p* ≤ 0.001; ** *p* ≤ 0.01; * *p* ≤ 0.05.

Expression changes of PPARγ (mRNA and protein) and miR-34a-5p in pAds, differentiated Ads and mature Ads cultured in normoglycemic and/or hyperglycemic conditions are depicted in Figure 5. While mRNA expression of PPARγ was not affected by achievement of differentiation, it declined in mature Ads compared with pAds (NNN vs N) (Figure 5a). Similarly, the lowest protein concentration of PPARγ during normoglycemic ADG was expressed in mature Ads (NNN vs N, NNN vs NN) (Figure 5b). Considering ADG in HG, the mRNA level of PPARγ was increased in mature Ads, as compared to both pAds (HHH vs H) and differentiated Ads (HHH vs HH) (Figure 5A). In contrast, we found a significant gradual reduction of protein levels of PPARγ during ADG in HG-treated cells (Figure 5b). We observed no significant changes of miR-34a-5p in normo-and hyperglycemic ADG (Figure 5c).

To conclude, while mRNA and protein expression data indicated downregulation of PPARγ during normoglycemic ADG, inconsistent mRNA and protein expression changes of PPARγ were reported during the HG-affected process. Based on the obtained results, no clear regulatory influence of miR-34a-5p on PPARγ can be implied during either type of ADG on its own.

#### 3.4.2. Chronic and Intermittent HG-Triggered Expression Changes

Considering stage I of cell culture, we found no significant differences in expression levels of both PPARγ protein levels and miR-34a-5p upon HG (H vs N) (Figure 5b,c). Nevertheless, the mRNA level of PPARγ exhibited a tendency towards downregulation in HG-affected pAds (H vs N, *p* = 0.0643) (Figure 5a). In differentiated Ads/stage II of cell culture, we observed no significant changes of mRNA levels of PPARγ in response to hyperglycemic stimulation (Figure 5a). However, we found PPARγ protein downregulation in chronically HG-treated differentiated Ads in comparison to ones treated with HG only during culture stage I(HH vs HN) along with the trend for corresponding upregulation of miR-34a-5p (HH vs HN, *p* = 0.0707) (Figure 5b,c). This could suggest a negative influence of miR-34a-5p on PPARγ expression during differentiation.

In stage III of culture, we observed trends and significant increases of mRNA and protein levels of PPARγ in cells treated solely with one HG stimulus (NNH, NHN, HNN) in comparison with normoglycemic ones (NNN) (mRNA-NNH vs NNN, *p* = 0.0583; protein-HNN vs NNN, *p* = 0.0862) (Figure 5a,b). Considering the impact of double and chronic exposure to HG versus NG, we found upregulation of mRNA levels of PPARγ only in the HHH and NHH variants, but no changes in PPARγ protein concentration (Figure 5a,b), which underscores the similarity of these variants. Importantly, we noted a significant downregulation of miR-34a-5p in cells exposed to single, double and chronic hyperglycemic stimulation, as compared to the untreated cells (NNN) (Figure 5c). This makes us speculate about possible negative regulatory impact of miR-34a-5p on expression of PPARγ in mature Ads upon HG.

Regarding the influence of chronic HG versus double HG hits, we observed no changes between mRNA and protein levels of PPARγ in the HHH versus the NHH (Figure 5a,b). However, comparison of HHH cells to HNH and HHN ones indicated an increase of mRNA level of PPARγ and a decrease of PPARγ protein concentration (Figure 5a,b). To further elucidate the impact of particular stage-specific HG hits, we compared expression profiles of cells treated with double HG stimulito ones exposed to single HG hits. Firstly, we found a decrease of mRNA and protein levels of PPARγ in the HHN variant in comparison with the NHN one (Figure 5a,b). The trend for PPARγ mRNA downregulation was also observed in the HHN versus the HNN (Figure 5a). In another variant with double hyperglycemic stimulation, NHH, we found upregulation of PPARγ mRNA and downregulation of protein level of PPARγ when compared to the NHN and NNH cells (Figure 5a,b). Finally, no changes of PPARγ protein concentration and reduction of PPARγ mRNA levels were reported in the HNH variant in relation to the HNN and NNH (Figure 5a,b). In contrast, all HG-treated types of mature Ads exhibited a similar expression change of miR-34a-5p, which made us speculate about the phenomenon of memorization of the effect mediated by HG (Figure 5c).

Results as a whole suggested that expression of PPARγ was sensitive to HG at each stage of visceral ADG, while changes of miR-34a-5p appeared only in mature Ads upon exposure to at least one HG hit introduced during a culture stage. Among the most intriguing observations one can enumerate were as follows:(i) PPARγ expression changes in the HHH cells were substantially different from all other HG-treated variants of mature Ads, expect for the NHH one. (ii) The potential mechanism of adaptation of PPARγ protein levels to chronic HG in mature Ads could be related to miR-34a-5p. (iii) There was a substantial impact by intermittent HG on mRNA and protein expression of PPARγ. (iv) The potential memorization of the impact of HG by miR-34a-5p could further suggest its significant biological role in mature Ads. Finally, due to the high level of similarity between results obtained for the HHH and NHH variants, we may also assume that the impact of HG during pAds proliferation might be the least significant for changes of PPARγ expression observed in visceral cells chronically exposed to HG.

## 4. Discussion

The main aim of our research was to evaluate changes in human visceral p/Ads exposed to intermittent and chronic HG, in regards to their morphology during ADG and the levels of ADG-related molecules, including miRNAs. To the best of our knowledge, this is the first study showing the effect of HG on morphological changes of p/Ads during visceral ADG. We found that HG markedly accelerated and intensified the process of HPA-v ADG in comparison with NG (Figure 1). Our studies revealed significant HG-triggered changes in the sizes of cells, which was associated with increased lipid accumulation (Figure 1). Chronic HG (HHH) triggered formation of the most increased volumes of lipid droplets among all of the other HG-exposed variants of mature Ads in comparison with the NNN ones. These results seem to suggest that the effect of accelerated differentiation appeared due to enhanced hypertrophy. This abnormal and premature enlargement of cells may lead to an inflammation state, which is one of the inseparable elements of T2DM. Considering intermittent HG, we could indeed remark that differentiated Ads exposed to single or double HG hits were substantially different from NN ones. Moreover, in mature Ads, it seems that the observed changes were dependent mainly on the length of exposure time to HG, rather than being connected with a specific stage of culture. This may suggest that the effect of HG is not completely reversible, but may be diminished by later normalization.

Results obtained using Raman spectroscopy and imaging clearly confirm that these techniques are able to visualize lipid structures in single cells using well-defined filters. Moreover, the analysis of average Raman spectra can be used to compare lipid content and compositions [32]. All results presented in Figure 2a,b showed that Raman spectroscopy is a powerful technique formonitoring epigenetic modifications of Ads at different levels of maturity. Imaging results (Figure 3) confirmed that the number of lipid droplets was higher for Ads cultured in HG (HHH).

Interestingly, Dentelli et al. reported that adipose stem cells (ASC) isolated from diabetic patients were more abundant, yet in a more de-differentiated state, than those originating from healthy controls [34]. Moreover, proliferation of ASCs isolated from the controls was increased in comparison to NG [34]. Another study showed that type I diabetic serum promotes pAds (Simpson-Golabi-Behmel syndrome (SGBS) cell line) proliferation and induces their differentiation, while high glucose has no impact on the above phenomena [35]. As T2DM is well-known to be accompanied by obesity, one may speculate that our results are indirectly supported by data obtained by Verboven et al. [36], who observed that the size of visceral Ads was increased in obese patients in comparison with lean subjects [36]. However, our results are not in agreement with the data obtained by several research teams, which showed no differences in TG (*triglycerides*) accumulation of pAds (SGBS cell line, isolated subcutaneous pAds or AT-derived stromal cells) differentiated in HG as compared to NG [25,35,37,38,39]. However, the observed differences may be associated with the biology of subcutaneous and visceral cells (i.e., the rate of lipolysis and lipid accumulation).

In our study, we showed that the relative mRNA expression of a crucial ADG marker, CEBPα, was higher after completion of the differentiation process, with a marked decrease after the maturation stage under normoglycemic conditions. Our results remain in line with previous research that showed a substantial increase of expression of this marker during ADG [40,41,42]. Contrary to expectations, upregulation of mRNA of CEBPα appeared only at the maturation stage in cells cultured in chronic HG. What is more, it was recently found by Acosta et al. that the expression of C/EBPα correlated negatively with fat cell size [43]. This was also partially confirmed by our observations obtained with BODIPY505/515 staining and expression analysis, especially in cells treated with HG during the whole culture. In our study, Ads differentiated in chronic HG conditions showed downregulation of mRNA and protein of C/EBPα in comparison to the NN, which may be supported by another study [44]. Namely, in the group of overweight and obese, the expression of C/EBPα was lower in comparison with the normal-weight group. Our data also correlate favorably with results obtained by Andersen et al. [45]. Specifically, in visceral Ads, mRNA level of C/EBPα was downregulated in obese patients with T2DM more than in lean patients. Regarding C/EBPα protein, we observed that expression level appeared to be gradually increased during ADG, both in chronic NG and HG, which is thoroughly in line with previous research [42]. However, it was more marked upon NG, suggesting that high glucose intensifies and accelerates ADG, but not via increasing the level of C/EBPα.

Our study provides an insight into ADG during intermittent HG. The obtained results, both from mRNA and protein, imply that the expression level of C/EBPα is more dependent on stage-specific events than the length of the period of hyperglycemic exposure. Curiously, we observed that later normalization of glycemia after HG hits did not always result in equalization of mRNA and protein expression levels with ones obtained in cells treated with NG during the whole culture. In our view, these results indicate that visceral adipocytes may be the source of metabolic memory. This remains in line with data provided by Andersen et al. [45], and partially supports our earlier findings [29].

We examined miR-137-3p, which is considered to target C/EBPα and be an ADG inhibitor. The expression pattern of miR-137-3p obtained in our study did not appear to suggest any effect on expression of C/EBPα at either the mRNA or protein level. In the light of previous research, it seems surprising that we did not find any statistically significant changes during ADG or upon HG. Ortega et al. reported the slight downregulation of miR-137-3p in subcutaneous pAds and mature Ads from obese subjects in comparison with lean ones [46]. Moreover, Shin et al. revealed that overexpressed miR-137-3p elicits an inhibitory impact of proliferation and differentiation of human adipose tissue-derived stromal stem cells (hADSCs) [28]. However, it must be taken into account that, in contrast with the aforementioned studies, our study was conducted on visceral Ads. It seems that this molecule could be considered as a marker of adipogenesis in subcutaneous AT [46] and AT stromal cells [28], but not in visceral Ads.

Considering PPARγ expression changes, we found a decrease of its mRNA and protein in mature Ads during ADG in NG. However, other studies have shown that PPARγ is steadily increased during ADG [25,40,47]. During ADG in HG, we observed upregulation of PPARγ mRNA and downregulation of its protein in mature Ads. In the most similar published paper, a 14-day-long ADG of subcutaneous abdominal pAds in 25mM glucose conditions showed the gradual elevation of mRNA and protein levels of PPARγ; yet, as reported in our study, no differences were recorded between ADG in NG and HG [25]. As we confirmed the achievement of the ADG process in our cell model, we speculate that the obtained results may be connected with specificity of HPA-v cells.

In the case of stage-specific chronic HG exposure, we did not observe any changes of PPARγ protein concentration (HHH vs NNN, HH vs NN, H vs N). These results can be cautiously compared to ones obtained by Peshadry et al., who showed no differences between Ads differentiated in NG and HG, as well as by Andersen et al. [25,45], in which study visceral pAds were isolated from lean, obese and obese with T2DM patients, but showed no differences in PPARγ level by the third day of differentiation [45]. In contrast, we determined the increase of the mRNA level of PPARγ in the HHH Ads as compared to NNN ones. Thus, it is possible that downregulation of miR-34a-5p triggered the upregulation of PPARγ mRNA.

In the case of variable glycemic conditions, mRNA and protein levels of PPARγ were the most significantly affected in mature Ads. This may imply that exposure to HG is relevant during proliferation and differentiation, yet its impact might be the most pronouncedly reflected in mature Ads. The sensitivity of PPARγ to variable glycemia might also suggest its biological roles other than participation in ADG progression, such as lipid metabolism, insulin sensitivity, oxidative stress and inflammation [48].

As mentioned in the above section, we cannot exclude that miR-34a-5p affects expression of PPARγ in visceral Ads upon HG. While we did not find changes of miR-34a-5p during ADG, Ortega et al. showed its upregulation during subcutaneous ADG [46]. In the current study, we observed similar HG-triggered expression levels of miR-34a-5p in all variants of mature Ads in comparison with the NNN, suggesting that at least one HG hit was sufficient for its expression decline. miR-34a-5p changes were determined as irreversible despite NG restoration in diabetic mice hearts, indicating involvement in metabolic memory [49]. Moreover, HG elevated miR-34a-5p to trigger apoptosis of cardiomyocytes, which may suggest that miR-34a-5p downregulation in mature Ads possibly provides protection from cellular death [50]. miR-34a-5p is tightly associated with diabetes-related genes such as p53 and Sirt1, as well as lipid metabolism [51,52].

The current study may provide some novel information associated with metabolic memory, an epigenetics-underlain phenomenon of preserving the effect of HG and the development of diabetic complications. Recent data indicate that not only endothelial cells, but also Ads, myocardial or skeletal muscle cells, may express signs of memorization of the impact of HG or TNF-α exposure [29,45,49,53]. Although this hypothesis needs further study, one should also take into account common progenitors of endothelial cells and Ads [54]. Our previous study on the same cell culture model suggested memorization of HG via miRNAs in differentiated and mature Ads [29].

Considering strong and weak aspects of our study, several issues should be recognized. Firstly, to the best of our knowledge, this was the first study presenting morphological changes during human visceral ADG conducted under variable glycemic conditions. To evaluate the effect of chronic and intermittent HG, we determined changes in crucial adipogenic markers at three stages of ADG. Importantly, the majority of currently available data regarding ADG was derived from mice 3T3-L1 cells, involving only proliferation and differentiation stages. Thus, the novelty of our data is also associated with the usage of human visceral cells, HPA-v. To identify miRNAs targeting PPARγ and C/EBPα, we implemented a comprehensive approach involving miRNA screening analysis of several hundred molecules [29], followed by bioinformatics analysis and literature search. Moreover, the reference genes used for mRNA and miRNA expression profiling where chosen experimentally. Although we performed in vitro studies, variants with intermittent glycemia may reflect (to some limited extent) the clinical situations of patients experiencing glucose fluctuations, such as poorly controlled or undiagnosed diabetes with postprandial glucose levels that are too high. Considering flaws, we did not experimentally confirm the effect of miRNAs on changes of ADG markers or the biological relevance of studied molecules using loss or gain of function techniques. Secondly, our study provided data regarding only one epigenetic mechanism, including miRNAs.

## 5. Conclusions

Taken together, we found that both chronic and intermittent HG exert an influence on visceral ADG. HG evokes changes in the morphology of Ads, as well as the expression of PPARγ and C/EBPα. Our results indicate that the changes in PPARγ may be partially evoked by miR-34a-5p. Moreover, intermittent HG induces changes that are not always fully reversible when compared with NG-cultured cells, which constitutes an intriguing finding concerning the biology of Ads and T2DM pathogenesis.

## Figures and Tables

**Table 1:** Cell culture variants of HPA-v.

I Culture Stage. Proliferation of PAds (5 Days)	II Culture Stage Differentiation (12 Days)	III Culture Stage Maturation of Ads (6 Days)
**N**	-	-
**H**	-	-
**N**	N	-
**N**	H	-
**H**	N	-
**H**	H	-
**N**	N	N
**N**	N	H
**N**	H	N
**H**	N	N
**H**	H	H
**H**	N	H
**H**	H	N
**N**	H	H

N: normoglycemic culture conditions (basal medium containing approximately 5.5mM of glucose).H: hyperglycemic culture conditions (medium-supplemented to obtain 30mMd-(+)-glucose concentration).pAds: preadipocytes. Ads: adipocytes. “-”: cells not cultured in a particular step.

## References

[1] Ouchi N., Parker J.L., Lugus J.J., Walsh K. (2011). Adipokines in inflammation and metabolic disease. Nat. Rev. Immunol..

[2] Ahima R.S., Flier J.S. (2000). Adipose Tissue as an Endocrine Organ. Trends Endocrinol. Metab..

[3] Frühbeck G., Gómez-Ambrosi J., Muruzábal F.J., Burrell M.A. (2001). The adipocyte: A model for integration of endocrine and metabolic signaling in energy metabolism regulation. Am. J. Physiol. Metab..

[4] Visser M., Bouter L., McQuillan G.M., Wener M.H., Harris T.B. (1999). Elevated C-Reactive Protein Levels in Overweight and Obese Adults. JAMA.

[5] Pradhan A.D., Manson J.E., Rifai N., Buring J.E., Ridker P.M. (2001). C-Reactive Protein, Interleukin 6, and Risk of Developing Type 2 Diabetes Mellitus. JAMA.

[6] Avram M.M., Avram A.S., James W.D. (2007). Subcutaneous fat in normal and diseased states 3. Adipogenesis: From stem cell to fat cell. J. Am. Acad. Dermatol..

[7] Lowe C.E., O’Rahilly S., Rochford J. (2011). Adipogenesis at a glance. J. Cell Sci..

[8] Bost F., Aouadi M., Caron L., Binétruy B. (2005). The role of MAPKs in adipocyte differentiation and obesity. Biochimie.

[9] Farmer S.R. (2005). Regulation of PPARgamma activity during adipogenesis. Int. J.Obes. (Lond.).

[10] Wu Z., Rosen E.D., Brun R., Hauser S., Adelmant G., Troy A.E., McKeon C., Darlington G.J., Spiegelman B.M. (1999). Cross-regulation of C/EBP alpha and PPAR gamma controls the transcriptional pathway of adipogenesis and insulin sensitivity. Mol. Cell.

[11] Forman B.M., Tontonoz P., Chen J., Brun R.P., Spiegelman B.M., Evans R.M. (1995). 15-Deoxy-delta 12, 14-prostaglandin J2 is a ligand for the adipocyte determination factor PPAR gamma. Cell.

[12] Schoonjans K., Staels B., Auwerx J. (1996). Role of the peroxisome proliferator-activated receptor (PPAR) in mediating the effects of fibrates and fatty acids on gene expression. J. Lipid Res..

[13] Braissant O., Foufelle F., Scotto C., Dauça M., Wahli W. (1996). Differential expression of peroxisome proliferator-activated receptors (PPARs): Tissue distribution of PPAR-alpha, -beta, and -gamma in the adult rat. Endocrinology.

[14] Lin F.T., MacDougald O.A., Diehl A.M., Lane M.D. (1993). A 30-kDa alternative translation product of the CCAAT/enhancer binding protein alpha message: Transcriptional activator lacking antimitotic activity. Proc. Natl. Acad. Sci. USA.

[15] Legraverend C., Antonson P., Flodby P., Xanthopoulos K.G. (1993). High level activity of the mouse CCAAT/enhancer binding protein (C/EBP alpha) gene promoter involves autoregulation and several ubiquitous transcription factors. Nucleic Acids Res..

[16] Chapman A.B., Knight D.M., Dieckmann B.S., Ringold G.M. (1984). Analysis of gene expression during differentiation of adipogenic cells in culture and hormonal control of the developmental program. J. Biol. Chem..

[17] Malodobra-Mazur M., Dziewulska A., Kozinski K., Dobrzyn P., Kolczyńska K., Janikiewicz J., Dobrzyn A. (2014). Stearoyl-CoA desaturase regulates inflammatory gene expression by changing DNA methylation level in 3T3 adipocytes. Int. J. Biochem. Cell Biol..

[18] Steger D.J., Grant G.R., Schupp M., Tomaru T., Lefterova M.I., Schug J., Manduchi E., Stoeckert C.J., Lazar M.A. (2010). Propagation of adipogenic signals through an epigenomic transition state. Genes Dev..

[19] Esau C., Kang X., Peralta E., Hanson E., Marcusson E.G., Ravichandran L.V., Sun Y., Koo S., Perera R.J., Jain R. (2004). MicroRNA-143 Regulates Adipocyte Differentiation. J. Biol. Chem..

[20] Ambros V. (2004). The functions of animal microRNAs. Nature.

[21] Zamore P.D. (2005). Ribo-gnome: The Big World of Small RNAs. Science.

[22] Aguiari P., Leo S., Zavan B., Vindigni V., Rimessi A., Bianchi K., Franzin C., Cortivo R., Rossato M., Vettor R. (2008). High glucose induces adipogenic differentiation of muscle-derived stem cells. Proc. Natl. Acad. Sci. USA.

[23] Wang W., Zhang X., Zheng J., Yang J. (2010). High glucose stimulates adipogenic and inhibits osteogenic differentiation in MG-63 cells through cAMP/protein kinase A/extracellular signal-regulated kinase pathway. Mol. Cell. Biochem..

[24] Zhang Y., Yang J.H. (2013). Activation of the PI 3 K/A kt pathway by oxidative stress mediates high glucose-induced increase of adipogenic differentiation in primary rat osteoblasts. J. Cell. Biochem..

[25] Peshdary V., Gagnon A., Sorisky A. (2016). Effect of High Glucose Concentration on Human Preadipocytes and Their Response to Macrophage-Conditioned Medium. Can. J. Diabetes.

[26] Sorisky A. (2017). Effect of High Glucose Levels on White Adipose Cells and Adipokines—Fuel for the Fire. Int. J. Mol. Sci..

[27] Ding J., Li M., Wan X., Jin X., Chen S., Yu C., Li Y. (2015). Effect of miR-34a in regulating steatosis by targeting PPARα expression in nonalcoholic fatty liver disease. Sci. Rep..

[28] Shin K.K., Kim Y.S., Kim J.Y., Bae Y.C., Jung J.S. (2014). miR-137 Controls Proliferation and Differentiation of Human Adipose Tissue Stromal Cells. Cell. Physiol. Biochem..

[29] Strycharz J., Świderska E., Wróblewski A., Podolska M., Czarny P., Szemraj J., Balcerczyk A., Drzewoski J., Kasznicki J., Śliwińska A. (2018). Hyperglycemia Affects miRNAs Expression Pattern during Adipogenesis of Human Visceral Adipocytes—Is Memorization Involved?. Nutrients.

[30] Xie F., Xiao P., Chen D., Xu L., Zhang B. (2012). miRDeepFinder: A miRNA analysis tool for deep sequencing of plant small RNAs. Plant Mol. Biol..

[31] Talari A.C.S., Movasaghi Z., Rehman S., Rehman I.U. (2015). Raman spectroscopy of biological tissues. Appl. Spectrosc. Rev..

[32] Brozek-Pluska B., Kopeć M., Abramczyk H. (2016). Development of a new diagnostic Raman method for monitoring epigenetic modifications in the cancer cells of human breast tissue. Anal. Methods.

[33] Rygula A., Majzner K., Marzec K.M., Kaczor A., Pilarczyk M., Baranska M. (2013). Raman spectroscopy of proteins: A review. J. Raman Spectrosc..

[34] Dentelli P., Barale C., Togliatto G., Trombetta A., Olgasi C., Gili M., Riganti C., Toppino M., Brizzi M.F. (2013). A diabetic milieu promotes OCT4 and NANOG production in human visceral-derived adipose stem cells. Diabetologia.

[35] Stuart A.A.V., Schipper H.S., Tasdelen I., Egan D.A., Prakken B.J., Kalkhoven E., De Jager W. (2012). Altered Plasma Adipokine Levels and in Vitro Adipocyte Differentiation in Pediatric Type 1 Diabetes. J. Clin. Endocrinol. Metab..

[36] Verboven K., Wouters K., Gaens K., Hansen D., Bijnen M., Wetzels S., Stehouwer C.D., Goossens G.H., Schalkwijk C.G., Blaak E.E. (2018). Abdominal subcutaneous and visceral adipocyte size, lipolysis and inflammation relate to insulin resistance in male obese humans. Sci. Rep..

[37] Rønningen T., Shah A., Reiner A.H., Collas P., Moskaug J. (2015). Øivind Epigenetic priming of inflammatory response genes by high glucose in adipose progenitor cells. Biochem. Biophys. Res. Commun..

[38] Collins J.M., Neville M.J., Pinnick K.E., Hodson L., Ruyter B., Van Dijk T.H., Reijngoud D.-J., Fielding M.D., Frayn K.N. (2011). De novo lipogenesis in the differentiating human adipocyte can provide all fatty acids necessary for maturation. J. Lipid Res..

[39] Cheng N.C., Hsieh T.Y., Lai H.S., Young T.H. (2016). High glucose-induced reactive oxygen species generation promotes stemness in human adipose-derived stem cells. Cytotherapy.

[40] Mandrup S., Lane M.D. (1997). Regulating adipogenesis. J. Biol. Chem..

[41] Darlington G.J., Ross S.E., MacDougald O. (1998). The Role of C/EBP Genes in Adipocyte Differentiation. J. Biol. Chem..

[42] Lane M., Tang Q.-Q., Jiang M.-S. (1999). Role of the CCAAT Enhancer Binding Proteins (C/EBPs) in Adipocyte Differentiation. Biochem. Biophys. Res. Commun..

[43] Acosta J.R., Douagi I., Andersson D.P., Bäckdahl J., Rydén M., Arner P., Laurencikiene J. (2016). Increased fat cell size: A major phenotype of subcutaneous white adipose tissue in non-obese individuals with type 2 diabetes. Diabetologia.

[44] Matulewicz N., Stefanowicz M., Nikołajuk A., Karczewska-Kupczewska M. (2017). Markers of adipogenesis, but not inflammation in adipose tissue, are independently related to insulin sensitivity. J. Clin. Endocrinol. Metab..

[45] Andersen E., Ingerslev L.R., Fabre O., Donkin I., Altıntaş A., Versteyhe S., Bisgaard T., Kristiansen V.B., Simar D., Barrès R. (2019). Preadipocytes from obese humans with type 2 diabetes are epigenetically reprogrammed at genes controlling adipose tissue function. Int. J. Obes..

[46] Ortega F.J., Moreno-Navarrete J.M., Pardo G., Sabater M., Hummel M., Ferrer A., Rodriguez-Hermosa J.I., Ruiz B., Ricart W., Peral B. (2010). MiRNA Expression Profile of Human Subcutaneous Adipose and during Adipocyte Differentiation. PLoS ONE.

[47] Strakovsky R.S., Lezmi S., Shkoda I., Flaws J.A., Helferich W.G., Pan Y.X. (2015). In Utero Growth Restriction and Catch-up Adipogenesis After Developmental Di (2-ethylhexyl) Phthalate (DEHP) Exposure Cause Glucose Intolerance in Adult Male Rats Following a High-fat Dietary Challenge. J. Nutr. Biochem..

[48] Janani C., Kumari B.R. (2015). PPAR gamma gene–A review. DiabetesMetab. Syndr..

[49] Costantino S., Paneni F., Cosentino F., Lüscher T.F. (2015). MicroRNA profiling unveils hyperglycaemic memory in the diabetic heart. Eur. Hear. J..

[50] Zhao F., Li B., Wei Y.Z., Zhou B., Wang H., Chen M., Gan X.D., Wang Z.H., Xiong S.X. (2013). MicroRNA-34a regulates high glucose-induced apoptosis in H9c2 cardiomyocytes. Acta Acad. Med. Wuhan.

[51] Wu J., Liang W., Tian Y., Ma F., Huang W., Jia Y., Jiang Z., Wu H. (2019). Inhibition of P53/miR-34a improves diabetic endothelial dysfunction via activation of SIRT1. J. Cell. Mol. Med..

[52] Yang Z., Cappello T., Wang L. (2015). Emerging role of microRNAs in lipid metabolism. Acta Pharm. Sin. B.

[53] Sharples A.P., Stewart C.E., Seaborne R.A. (2016). Does skeletal muscle have an ‘epi’-memory? The role of epigenetics in nutritional programming, metabolic disease, aging and exercise. Aging Cell.

[54] Tran K.V., Gealekman O., Frontini A., Zingaretti M.C., Morroni M., Giordano A., Smorlesi A., Perugini J., De Matteis R., Sbarbati A. (2012). The vascular endothelium of the adipose tissue give rise to both white and brown fat cells. Cell Metab..

